# Book Notices

**Published:** 1868-12

**Authors:** 


### Book Notices.

---

A Manual of the Dissection of the Human Body. By LUTHER HOLDEN, F.R.C.S., Assistant Surgeon of, and Lecturer on Anatomy at, St. Bartholomew's Hospital, London. With Notes and Additions. By ERSKINE MASON, M.D., Demonstrator of Anatomy at the College of Physicians and Surgeons, and Surgeon to the Charity Hospital, New York. Illustrated with numerous Wood Engravings. New York: Robert M. De Witt, No. 13 Frankfort Street. 1868. Pp. 588.

This is a new and enlarged edition of the well-known Holden's Dissector. It is published in excellent style, on good type and paper, with excellent illustrations. The additions made by the American Editor are numerous and valuable. It is one of the most convenient and accurate guides to the study of practical anatomy that we have seen.

---

A Treatise on the Principles and Practice of Medicine: Designed for the Use of Practitioners and Students of Medicine. By AUSTIN FLINT, M.D., Professor of the Principles and Practice of Medicine in the Bellevue Hospital Medical College, Fellow of the New York Academy of Medicine, etc. Third Edition, thoroughly revised. Philadelphia: Henry C. Lea. 1868.

Dr. Flint's Treatise has now been before the public nearly three years, and the rapid exhaustion of the two first editions shows that its merits have been appreciated. The present edition contains evidence of having been carefully revised by the author, and presents the present state of practical medicine in as interesting and concise a form as is possible. The work is substantially bound, and contains 1002 closely-printed pages. For sale by S. C. Griggs & Co., Chicago. Price, $7.00.

---

Outlines of Physiology, Human and Comparative. By JOHN MARSHALL, F.R.S., Professor of Surgery in University College, London; Surgeon to the University College Hospital. With Additions. By FRANCIS G. SMITH, M.D., Professor of the Institutes of Medicine in the University of Pennsylvania. Illustrated by numerous Woodcuts. Philadelphia: Henry C. Lea. 1868.

The author of this work has aimed to condense within the limits of a convenient text-book a complete summary of the present state of knowledge on the subject of both human and comparative physiology. In this he has succeeded well. The additions by the American Editor are numerous, and increase the value of the work. It will be found one of the most convenient and useful works on physiology that we possess. The work is substantially bound, and contains over 1000 pages. For sale by W. B. Keen & Co., Chicago.

---

The Medical Formulary: Being a Collection of Prescriptions [illegible]ived from the Writings and Practice of many of the most eminent Physicians in America and Europe; together with the usual Dietetic Preparations and Antidotes for Poisons. To which is added an Appendix, on the Endermic Use of Medicines, and on the Use of Ether and Chloroform. The whole accompanied with a few brief Pharmaceutical and Medical Observations. By BENJ. ELLIS, M.D., late Professor of Materia Medica and Pharmacy in the Philadelphia College of Pharmacy. Twelfth Edition, carefully revised and much improved. By ALBERT H. SMITH, M.D., Fellow of the College of Physicians of Philadelphia; Lecturer on Obstetrics to the Philadelphia Lying-in Charity, etc., etc. Philadelphia: Henry C. Lea. 1868. Price, $3.00.

This is a neatly-bound octavo volume of 374 pages. Its contents are well indicated by the title-page. The most ardent admirer of formulæ will find here an abundance of samples for study.

---

Atlas of Venereal Diseases. By A. CULLERIER, Surgeon to the Hospital Du Midi; Member of the Surgical Society of Paris, etc., etc. Translated from the French, with Notes and Additions. By FREEMAN J. BUMSTEAD, M.D., Professor of Venereal Diseases in the College of Physicians and Surgeons, New York, etc. Philadelphia: Henry C. Lea. 1868. Part 5.

The fifth, and last, part of this valuable work has been received, and completes the volume. It is accompanied by a copious index, title-page, etc., and also a neat and substantial binding. This part is occupied chiefly with the consideration of Secondary and Tertiary Syphilis. The plates are executed in the best style; and the whole work does much credit to both authors and publishers. For sale by W. B. Keen & Co., 148 Lake Street, Chicago.

---

Doctor or Doctress? By SAMUEL GREGORY, A.M., M.D., Secretary of the New England Female Medical College.

We have received a little pamphlet with this title, advocating the use of the word *Doctress* to designate female physicians. We approve the plan. Let the female physician have a *feminine* title, by all means.

